# Supplementary material for: Decorative Chromium Coatings on Polycarbonate Substrate for the Automotive Industry
Source: Materials (Basel). 2023 Mar 14;16(6):2315. doi: 10.3390/ma16062315 (PMC10051204; doi:10.3390/ma16062315)
Supplement: Supplementary file 1 [file materials-16-02315-s001.zip › materials-2168388-supplementary.pdf]

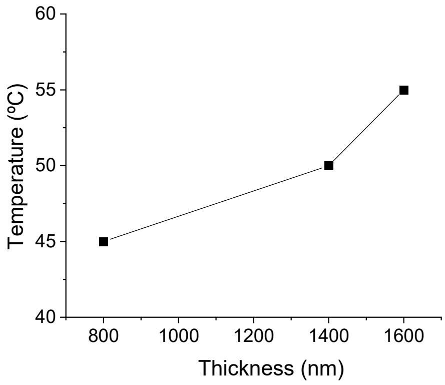

Figure S1. Substrate temperature as a function of Cr film thickness.

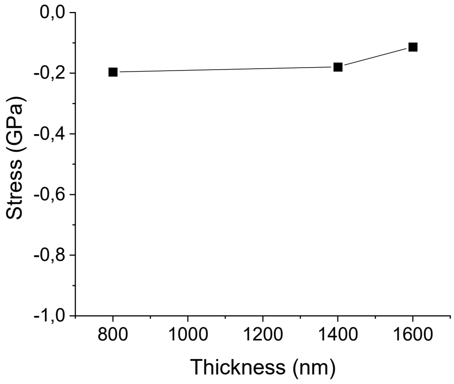

Figure S2. Evolution of residual stress with film thickness for Cr coatings deposited on Si.

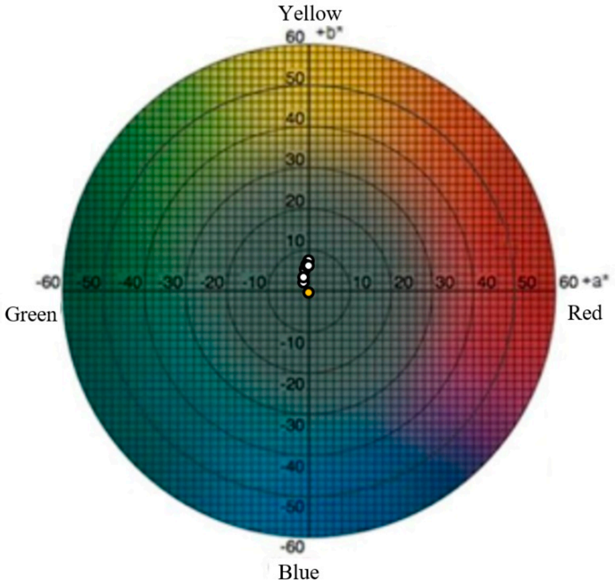

Figure S3. CIE-L*a*b* color diagram containing both experimental Cr film color coordinates (white points) and ideal Cr film color coordinates (yellow point). Adapted from [50].

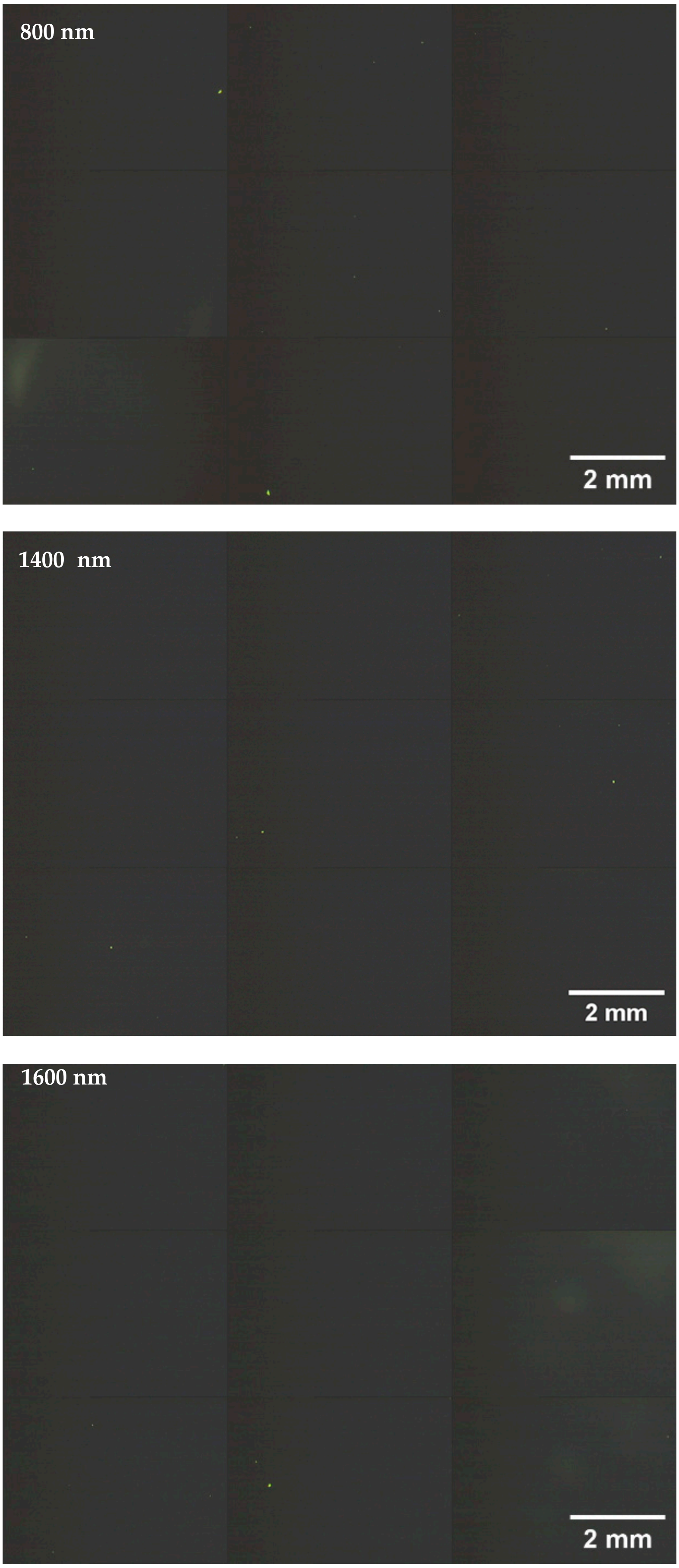

Figure S4. Microscopic images of Cr coatings on PCB samples using backlight mode for counting the number of pinholes.
